# The occurrence of ESBL-producing *Escherichia coli* carrying aminoglycoside resistance genes in urinary tract infections in Saudi Arabia

**DOI:** 10.1186/s12941-016-0177-6

**Published:** 2017-01-06

**Authors:** Essam J. Alyamani, Anamil M. Khiyami, Rayan Y. Booq, Majed A. Majrashi, Fayez S. Bahwerth, Elena Rechkina

**Affiliations:** 1 National Center for Biotechnology, King Abdulaziz City for Science and Technology, P.O. Box 6086, Riyadh, 11442 Saudi Arabia; 2 College of Medicine, Princess Nora Bint Abdul Rahman University, Riyadh, 12484 Saudi Arabia; 3 Hera Hospital, Makkah, Saudi Arabia; 4 ID Genomics, Seattle, WA USA

**Keywords:** ESBL, *E. coli*, *K. pneumonia*, Phenotyping, Genotyping, Saudi Arabia

## Abstract

**Background:**

The infection and prevalence of extended-spectrum β-lactamases (ESBLs) is a worldwide problem, and the presence of ESBLs varies between countries. In this study, we investigated the occurrence of plasmid-mediated ESBL/AmpC/carbapenemase/aminoglycoside resistance gene expression in *Escherichia coli* using phenotypic and genotypic techniques.

**Methods:**

A total of 58 *E. coli* isolates were collected from hospitals in the city of Makkah and screened for the production of ESBL/AmpC/carbapenemase/aminoglycoside resistance genes. All samples were subjected to phenotypic and genotypic analyses. The antibiotic susceptibility of the *E. coli* isolates was determined using the Vitek-2 system and the minimum inhibitory concentration (MIC) assay. Antimicrobial agents tested using the Vitek 2 system and MIC assay included the expanded-spectrum (or third-generation) cephalosporins (e.g., cefoxitin, cefepime, aztreonam, cefotaxime, ceftriaxone, and ceftazidime) and carbapenems (meropenem and imipenem). Reported positive isolates were investigated using genotyping technology (oligonucleotide microarray-based assay and PCR). The genotyping investigation was focused on ESBL variants and the AmpC, carbapenemase and aminoglycoside resistance genes*. E*. *coli* was phylogenetically grouped, and the clonality of the isolates was studied using multilocus sequence typing (MLST).

**Results:**

Our *E. coli* isolates exhibited different levels of resistance to ESBL drugs, including ampicillin (96.61%), cefoxitin (15.25%), ciprofloxacin (79.66%), cefepime (75.58%), aztreonam (89.83%), cefotaxime (76.27%), ceftazidime (81.36%), meropenem (0%) and imipenem (0%). Furthermore, the distribution of ESBL-producing *E. coli* was consistent with the data obtained using an oligonucleotide microarray-based assay and PCR genotyping against genes associated with β-lactam resistance. ST131 was the dominant sequence type lineage of the isolates and was the most uropathogenic *E. coli* lineage. The *E. coli* isolates also carried aminoglycoside resistance genes.

**Conclusions:**

The evolution and prevalence of ESBL-producing *E. coli* may be rapidly accelerating in Saudi Arabia due to the high visitation seasons (especially to the city of Makkah). The health authority in Saudi Arabia should monitor the level of drug resistance in all general hospitals to reduce the increasing trend of microbial drug resistance and the impact on patient therapy.

## Background

Gram-negative bacteria that produce β-lactamases are a major concern in healthcare due to their ability to spread globally [1]. β-lactamases are produced by bacteria to hydrolyze the β-lactam ring in antibiotics, which results in ineffective drug treatment. Extended-spectrum β lactamases (ESBLs) are a major group of enzymes that confer resistance to several generations of β-lactam antibiotics, including third-generation cephalosporins [2, 3]. ESBL-producing Gram-negative bacteria are thought to be an important reason for cephalosporin therapy failure [4, 5]. Therefore, these bacteria should be monitored and reported by clinical laboratories to minimize their impact on patient therapy. ESBL resistance genes are primarily carried by plasmids. Plasmids may also carry genes encoding resistance to other antibiotic classes, such as ampA, ampC, aminoglycosides, chloramphenicol, macrolides, or quinolone. Therefore, treatment options are limited for bacteria that produce ESBLs due to the multiple resistance genes encoded in the plasmids. ESBL genes that are primarily encoded in plasmids include TEM, SHV, and CTX-M. Multiple variants of each gene are produced by altering the configuration of amino acids within the β-lactamase active site. Enterobacteriaceae, such as *Klebsiella pneumoniae* and *Escherichia coli*, are the major ESBL producers frequently isolated in clinical laboratories [6]. *Acinetobacter baumannii* is also an important ESBL-producing bacteria that has been reported globally [7]. Newer antibiotic classes, such as carbapenems, have been introduced by the pharmaceutical industry as treatment options for infections caused by ESBL-producing bacteria. Nevertheless, carbapenemase-producing bacteria have also been documented [8, 9]. Due to the increasing threat of multidrug-resistant bacteria, laboratory personnel, physicians, and clinical practitioners should implement a program to detect and report ESBLs as part of their infection control to limit the therapeutic failures caused by ESBL-producing bacteria. Molecular genotyping has been used concurrently with phenotyping techniques to detect and confirm antimicrobial drug resistance data and to detect Gram-negative ESBL producing bacteria [10]. PCR, multiplex PCR and oligonucleotide microarray-based assays have been developed and used to monitor the emergence of ESBLs and many other drug-resistant genes from *E. coli*, *K. pneumonia* and *A. baumannii* [11–15]. Strain characterization by multilocus sequence typing (MLST) is the method of choice in many clinical and research laboratories due to its high discriminatory power [16, 17]. The increased prevalence of ESBLs is being monitored and reported globally. Due to a lack of a solid data regarding the emergence of ESBLs from major Saudi general hospitals in the region of Makkah, this study reports the characterization of drug resistance genes for 58 *E. coli* isolates from an in-patient ward using phenotypic and genotypic approaches. Understanding the phenotypic and molecular nature of Enterobacteriaceae during the busy visitation Hajj season (pilgrimage season) in the city of Makkah, Saudi Arabia, is essential to reducing ESBL-strains prevalence.

## Methods

### Bacteriological culture

A total of 58 bacterial isolates were collected from two different general hospitals in the city of Makkah during the 2014–2015 Hajj (pilgrimage) season from patients with urinary tract infections. The bacterial isolates were phenotypically and genotypically investigated in microbiology laboratories at the King Abdulaziz City for Science and Technology (KACST). Single pure *E. coli* colonies were obtained from the all isolates. The bacteria were isolated from urine specimens using the clean-catch midstream urine sampling technique. Urine samples were inoculated using a calibrated 0.01 mL urine plastic loop on 5% sheep blood agar and MacConkey agar plates. The plates were incubated at 37 °C for 24 h. Urine samples were considered positive for UTIs if the number of colonies equaled or exceeded 10^5^ CFU/mL. Gram staining was performed to identify urine specimens that contained more than 10^5^ colony forming units (CFU)/mL of bacteria. A drop of well-mixed urine was fixed on a glass slide, stained, and examined under oil immersion (1000×) for the presence of at least one organism per oil immersion field.

### Bacterial identification and antibiotic susceptibility testing

Bacterial identities were confirmed with a Vitek 2 GN ID card using the Vitek 2 system (bioMérieux, Marcy I’Etoile, France). Antibiotic susceptibility testing (AST) was completed using Vitek 2 cards (AST-N292) according to the manufacturer’s recommendations (bioMérieux, Marcy I’Etoile, France). The Vitek ESBL susceptibility tests were interpreted according to the Clinical and Laboratory Standards Institute (CLSI) criteria using the Vitek system. *E. coli* ATCC 25922 was included in each Vitek testing step for quality control. The minimum inhibitory concentrations (MICs) were determined in Mueller–Hinton broth using microdilution plates [18]. The MICs of cefoxitin, ciprofloxacin, aztreonam, ceftazidime, meropenem, cefepime, cefotaxime, imipenem and ampicillin for all *E. coli* isolates were interpreted according to previous protocols (CLSI, 2014) [18]. Serial dilutions of the nine drugs were prepared (0.5, 1, 2, 4, 8, 16, 32, 64, 128, 256, 512 and 1024 µg/mL) in Mueller–Hinton broth and added to 96-well plates. Then, the bacterial suspensions were added to each well to achieve a final inoculum of 5×10^5^ CFU/mL. The endpoint MIC was read visually and by spectrophotometer at 600 nm. The lowest concentration of antibiotic that inhibited visible bacterial growth after 24 h at 37 °C was defined as the MIC.

### Amplification and sequencing of the 16S rRNA gene

The *E. coli* identity was confirmed using PCR and sequencing of the 16S rDNA gene [19–21]. Illustra PuReTaq Ready-To-Go PCR beads were used in the PCR reaction (GE Health Biosciences, USA). The 25-μL reaction was set up as follows: 2 μL of 10 pmol of each forward and reverse primer (IDT, Integrated DNA Technologies) were used with the 8-forward primer (AGA GTT TGA TCC TGG CTC AG) and 805-reverse primer (GAC TAC CAG GGT ATC TAA TC) [22]. Exactly 1 μL of DNA template was added to the beads, and the reaction was completed using 22 μL of nuclease-free ddH_2_O (Promega). The amplification cycling conditions were 3 min for the initial incubation at 95 °C, followed by 35 cycles of 1 min at 95 °C, 1 min at 55 °C, and 2 min at 72 °C and a final extension at 72 °C for 7 min. All PCR amplicons were fractionated using 1% agarose gel electrophoresis prior to staining with ethidium bromide. Images were obtained with a gel documentation system under a UV transilluminator. The PCR amplicons were purified and sequenced in an ABI 3130 Genetic Analyzer (Life Technologies, Carlsbad, CA, USA) using the 16S rDNA forward primer with the ABI BigDye terminator cycle sequencing ready reaction kit according to the manufacturer’s recommendations (Applied Biosystem, Foster City, CA, USA). The sequencing data were analyzed using the basic local alignment search tool BLAST-n (http://www.ncbi.nlm.nih.gov/BLAST) or the RDP database [23]. Unequivocal identification was obtained using 16S ribosomal DNA sequences.

### Genetic background grouping of *E. coli* strains

The genetic background grouping of the *E. coli* isolates was explored. The grouping was performed based on Clermont phylo-typing method [24]. The majority of the pathogenic extra-intestinal *E. coli* strains belonged to groups B2. In contrast, the commensal *E. coli* strains belonged to groups A, B1, C, and F. The strains were subsequently phylogenetically inferred based on the presence or absence of the gene markers as follows: For group A, arpA+, chuA−, yjaA−, and TspE4.C2−, for group B1, arpA+, chuA−, yjaA−, and TspE4.C2+, for group F, arpA−, chuA+, yjaA−, and TspE4.C2−, for group B2, arpA−, chuA+, yjaA+, and TspE4.C2−, for group A or C, arpA+, chuA−, yjaA+, and TspE4.C2−, for group D or E, arpA+, chuA+, yjaA−, and TspE4.C2−. A standard 25-µL PCR reaction was used to investigate the genetic background of the *E. coli* strains used in our study. Illustra PuReTaq Ready-To-Go PCR beads were used in the PCR reaction (GE Health Biosciences, USA) as described by Clermont to amplify the arpA (400 bp) ChuA (288 bp), YjaA (211 bp), TspE4C2 (152 bp), and an internal control trpA (489 bp). Primers and PCR cycling conditions were described in (Table 1).

**Table 1:** Primer sequences and PCR conditions for quadruplex phylo-grouping of *E. coli*

Target gene	Primer sequence	Size (bp)	PCR conditions	Reference
arpA (phylogenetic grouping)	F (5-AACGCTATTCGCCAGCTTGC-3) R (5-TCTCCCCATACCGTACGCTA-3)	400	PCR reactions were performed in a total volume of 25 µL by ready-to go PCR beads using 20 pmol of each primers for all targets except arpA (40 pmol) and trpA (12 pmol). The cycling conditions were as follow: denaturation 4 min at 94 °C, 30 cycles of 5 s at 94 °C and 20 s at 59 °C, 20 s at 72 °C and a final extension step of 5 min at 72 °C	[24]
chuA (phylogenetic grouping)	F (5-GACGAACCAACGGTCAGGAT-3) R (5-TGCCGCCAGTACCAAAGACA-3)	288		
yjaA (phylogenetic grouping)	F (5-TGAAGTGTCAGGAGACGCTG-3) R (5-ATGGAGAATGCGTTCCTCAAC-3)	211		
TspE4C2 (phylogenetic grouping)	F (5-GAGTAATGTCGGGGCATTCA-3) R (5-GCGCCAACAAAGTATTACG-3)	152		
Internal control trpA	F (5-CGGCGATAAAGACATCTTCAC-3) R (5-GCAACGCGGCCTGGCGGAAG-3)	489		

### Detection of ESBL, ampC and carbapenemase genes

Global genotyping utilizing an oligonucleotide microarray-based assay and PCR genotyping were applied to identify and confirm the presence of drug resistance genes. For the microarray DNA analysis (Alere Technologies, Jena, Germany) [15], the ESBL/AmpC/carbapenemase genes were evaluated based on the manufacturer’s protocols (*bla*_*ACC*_, *bla*_*ACT*_, *bla*_*CMY*_, *bla*_*KHM*_, *bla*_*MOX*-*CMY9*_, *bla*_*CTX*-*M*-*1*_, *bla*_*CTX*-*M*-*15*_, *bla*_*CTX*-*M 2*_, *bla*_*CTX*-*M*-*8*_, *bla*_*CTX*-*M*-*9*_, *bla*_*CTX*-*M*-*26*_, *bla*_*DHA*-*1*_, *bla*_*FOX*_, *bla*_*GES*-*1*_, *bla*_*G1M*-*1*_, *bla*_*MI*-*3*_, *bla*_*IMP*_, *bla*_*KPC*_, *bla*_*LAP*-*1*_, *bla*_*LEN*-*1*_, *bla*_*MOX*_, *bla*_*OXA*-*1*_, *bla*_*OXA*-*2*_, *bla*_*OXA*-*7*_, *bla*_*OXA*-*9*_, *bla*_*OXA*-*23*_, *bla*_*OXA*-*23*_, *bla*_*OXA*-*40*_, *bla*_*OXA*-*48*_, *bla*_*OXA*-*51*_, *bla*_*OXA*-*54*_, *bla*_*OXA*-*55*_, *bla*_*OXA*-*58*_, *bla*_*OXA*-*60*_, *bla*_*PER1*_, *bla*_*PER2*_, *bla*_*PSE*-*1*_, *bla*_*SHF*-*1*_, *bla*_*SHV*_, *bla*_*SEM*-*1*_, *bla*_*SPM*-*1*_, *bla*_*TEM*_, *bla*_*VEB*-*1*_, and *bla*_*VIM*_). For the PCR analysis, DNA templates were obtained by boiling 500 µL of bacterial cells (OD_600_ = 1.0) in a sterile single 1.5 mL Eppendorf tube in a water bath at 100 °C. The DNA lysate was diluted 1:10 with ddH_2_O and frozen at −20 °C before use for bacterial genotyping confirmation with gene-specific primers (Table 2) [10]. The primers detected the β-lactamase genes and their variants (TEM, SHV, and CTX-M groups 1, 15, 2, 8, 9, and 26). The PCRs were performed using a 9800 Thermal Cycler (Applied Biosystem, USA) in a total volume of 25 µL containing 10 pmol of primers, 25 µmol of dNTPs, 10 µL of gDNA/plasmid lysate, 2.5 µL of 10Χ *Taq* buffer, 2.5 mM MgCl_2_ and 2.5 U of *Taq* polymerase. The cycling conditions used for the PCR were an initial denaturation at 94 °C for 10 min, 35 cycles of 94 °C for 40 s, 60 °C for 40 s and 72 °C for 1 min and a final extension step at 72 °C for 7 min [10]. All PCR amplicons were fractionated by 1% agarose gel electrophoresis prior to staining with ethidium bromide. Images were obtained with a gel documentation system under a UV transilluminator.

**Table 2:** Multiplex PCR-specific primers for ESBL gene detection in *Enterobacteriacaea*

Primer name	β-Lactamase target	Sequence (5′–3′)	Length (bases)	Amplicon size (bp)	Reference
Multiplex I TEM, SHV	TEM variants including TEM-1 and TEM-2	Multi- F CATTTCCGTGTCGCCCTTATTC Multi- R CGTTCATCCATAGTTGCCTGAC	22	800	[10]
	SHV variants including SHV-1	Multi-F AGCCGCTTGAGCAAATTAAAC Multi-R ATCCCGCAGATAAATCACCAC	21	713	
Multiplex II CTX-M group 1	Variants of CTX-M group 1 including CTX-M-1, CTX-M-3 and CTX-M-15	MultiCTX-M Gp1-F TTAGGAARTGTGCCGCTGYA MultiCTX-MGP1-R CGATATCGTTGGTGGTRCCAT	20	688	
			21		
Multiplex II CTX-M group 2	Variants of CTX-M group 2 including CTX-M-2	MultiCTX-MGp2-F CGTTAACGGCACGATGAC MultiCTXMGp2-R CGATATCGTTGGTGGTRCCA	18	404	
			21		
Multiplex II CTX-M group 9	Variants of CTX-M group 9 including CTX-M-9 and CTX-M-14	MultiCTX-MGp9-F TCAAGCCTGCCGATCTGGT MultiCTX-MGp9-R TGATTCTCGCCGCTGAAG	19	561	
			18		
Multiplex III CTX-M group 8/25	CTX-M-8, CTX-M-25, CTX-M-26 and CTX-M-39 to CTX-M-41	MultiCTX-MGg8/25-F AACRCRCAGACGCTCTAC MultiCTX-MGg8/25-R TCGAGCCGGAASGTGTYAT	18	326	
			19		

### Bacterial genotyping by multilocus sequence typing

Fifty-eight *E. coli* isolates were subjected to multilocus sequence typing (MLST) as previously described [25]. The MLST schemes used to type *E. coli* were conducted using internal fragments of the following seven housekeeping genes: *Adk* (adenylate kinase), *fumC* (fumarate hydratase), *gyrB* (DNA gyrase), *mdh* (malate dehydrogenase), *purA* (adenylosuccinate dehydrogenase), *icd* (isocitrate/isopropylmalate dehydrogenase), *recA* (ATP/GTP binding motif) (ID Genomics Inc, Seattle, WA, USA). The primers used for amplification and sequencing are illustrated in (Table 3) [25]. The PCR amplifications were performed using a 9800 Thermal Cycler (Applied Biosystem, USA) with the following conditions: initial denaturation at 94 °C for 7 min, followed by 35 cycles of denaturation at 94 °C for 30 s and an annealing temperature of 56 °C for 30 s, extension at 72 °C for 2 min and a final 7-min extension at 72 °C. The PCR amplicons were checked with 1% agarose gel electrophoresis prior to purification for sequencing. Forward and reverse sequencing reactions were performed for every isolate. Different allele sequences were assigned for each locus with an arbitrary allele number for identification. Each bacterial isolate was characterized by a pattern of numbers defining its sequence type (ST). The sequencing data of the MLST genes were analyzed using the *E. coli* MLST Database (Warwick Medical School, Coventry, UK database; http://mlst.warwick.ac.uk/mlst/dbs/Ecoli) [25]. The similarities of the allelic profiles were assessed using the Molecular Evolutionary Genetics Analysis (MEGA 6) software.

**Table 3:** Primers used to amplify and sequence the seven housekeeping genes in the *E. coli* isolates for the MLST analysis

Gene	Primer sequences	Usage
*adk*	F 5′-ATTCTGCTTGGCGCTCCGGG -3′ R 5′-CCGTCAACTTTCGCGTATTT-3′	Amp/seq
*fumC*	F 5′-TCACAGGTCGCCAGCGCTTC-3′ R 5′-GTACGCAGCGAAAAAGATTC-3′	Amp/seq
*gyrB*	F 5′-TCGGCGACACGGATGACGG-3′ R 5′-ATCAGGCCTTCACGCGCATC-3′	Amp/seq
*icd*	F 5′-ATGGAAAGTAAAGTAGTTGTTCCGGCACA-3′ R 5′-GGACGCAGCAGGATCTGTT-3′	Amp/seq
*mdh*	F 5′-ATGAAAGTCGCAGTCCTCGGCGCTGCTGGCGG-3′ R 5′-TTAACGAACTCCTGCCCCAGAGCGATATCTTTCTT-3′	Amp/seq
*purA*	F 5′-CGCGCTGATGAAAGAGATGA-3′ R 5′-CATACGGTAAGCCACGCAGA-3′	Amp/seq
*recA*	F 5′-CGCATTCGCTTTACCCTGACC-3′ R 5′-TCTCGATCAGCTTCTCTTTT-3′	Amp/seq

### Phylogenetic analysis

Phylogenetic trees based on the concatenated alleles of seven MLST genes were constructed using the Molecular Evolutionary Genetics Analysis (Mega6) software for the *E. coli* dataset. The maximum likelihood trees were based on neighbor-joining (NJ) starting trees with Nearest-Neighbor Interchange branch swapping. The tree stability was assessed using the bootstrap method [26].

## Results

### Bacterial identification and antibiotic susceptibility testing

**Fig. 1:**
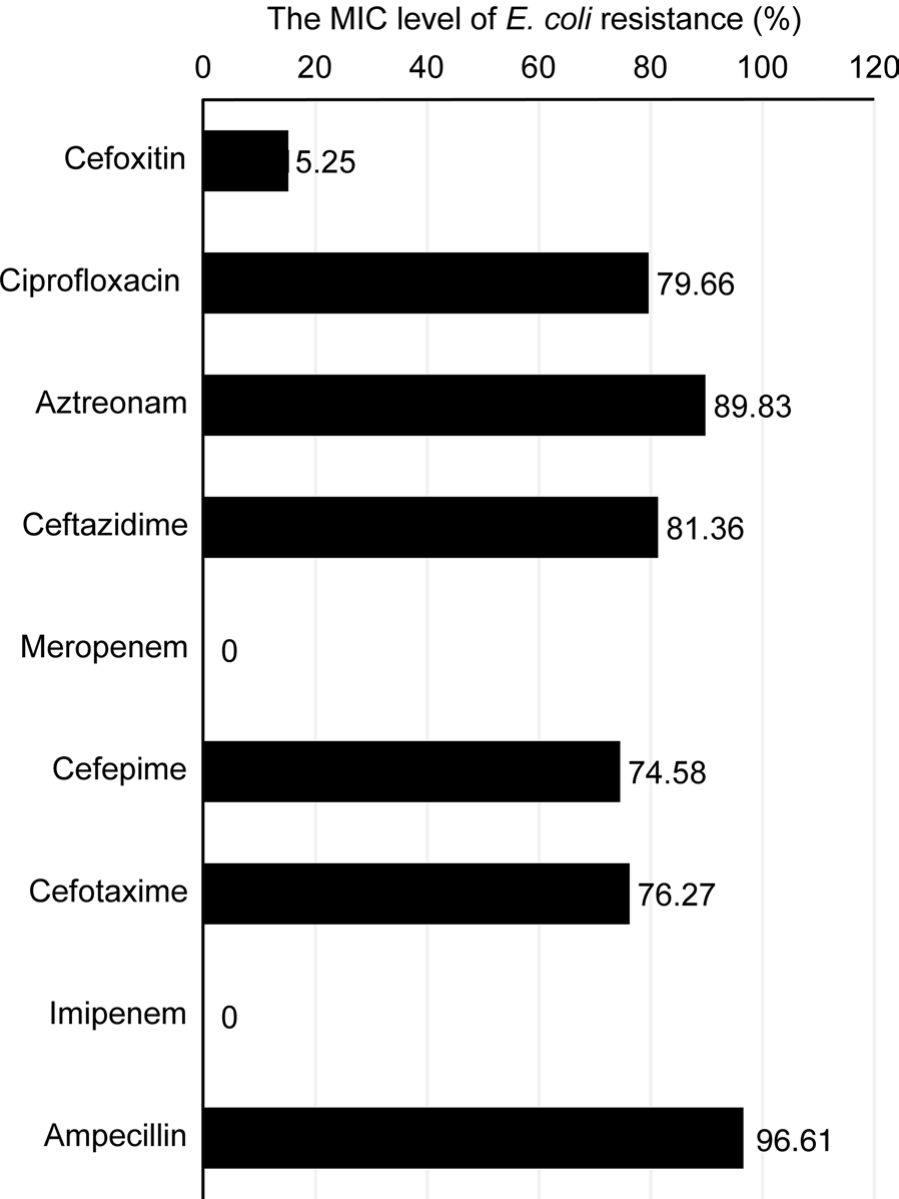
The level of *E. coli* resistance (in percentages) compared with the major ESBL drugs based on the MIC tests. No carbapenase phenotype was detected but Ciprofloxacin-resistance (fluoroquinolone resistance) was identified among the *E. coli* isolates

All obtained clinical *E. coli* isolates (n = 58) were phenotypically studied using the Vitek system and the microbroth dilution method to determine the MIC values against various antimicrobial drugs (cefoxitin, ciprofloxacin, aztreonam, ceftazidime, meropenem, cefepime, cefotaxime, imipenem and ampicillin), (Table 4). The greatest number of *E. coli* isolates was resistant to ampicillin (96.61%), followed by cefotaxime (76.27%), cefepime (74.58%), ceftazidime (81.36%), aztreonam (89.83%), and cefoxitin (15.25%). Meropenem and imipenem had rates of 0%, and the non-β-lactam ciprofloxacin had a rate of 79.66%. All *E. coli* isolates were sensitive to meropenem and imipenem (Fig. 1).

**Table 4:** Minimum inhibitory concentration (MIC) values of antimicrobial agents against ESBL-producing *E. coli*

Strains	Cefoxitin (µg/mL) R ≥ 32	Cefepime (µg/mL) R ≥ 16	Aztreonam (µg/mL) R ≥ 4	Ceftazidime (µg/mL) R ≥ 16	Cefotaxime (µg/mL) R ≥ 64	Ciprofloxacin (µg/mL) R ≥ 4	Meropenem (µg/mL) R ≥ 4	Imipenem (µg/mL) R ≥ 4	Ampecillin (µg/mL) R ≥ 32
	MIC	MIC	MIC	MIC	MIC	MIC	MIC	MIC	MIC
1059	8	32	256	128	1024	1024	<0.5	<0.5	>1024
1129	8	<0.5	<0.5	<0.5	<0.5	16	<0.5	<0.5	1024
1060	4	64	64	64	>1024	>1024	<0.5	<0.5	>1024
1097	4	64	128	32	>1024	<0.5	<0.5	<0.5	>1024
4110	8	8	128	16	64	32	<0.5	<0.5	1024
7055	1	16	<0.5	32	1024	1	<0.5	<0.5	>1024
1015	16	16	32	8	64	1024	<0.5	<0.5	512
2001	4	<0.5	128	64	1024	64	<0.5	<0.5	>1024
1114	8	256	512	128	>1024	128	<0.5	<0.5	>1024
1007	8	16	128	32	128	128	<0.5	<0.5	>1024
1111	256	128	128	128	>1024	64	<0.5	<0.5	>1024
1128	16	16	64	16	256	1024	<0.5	<0.5	1024
1005	4	4	64	16	1024	16	<0.5	<0.5	>1024
1116	8	16	32	8	>1024	64	<0.5	<0.5	>1024
1010	2	<0.5	<0.5	<0.5	2	32	<0.5	<0.5	>1024
1041	512	1024	>1024	1024	>1024	1024	<0.5	1	>1024
1089	8	128	128	32	1024	64	<0.5	<0.5	>1024
1031	16	32	128	32	256	32	<0.5	<0.5	>1024
1055	16	256	256	256	>1024	1024	<0.5	<0.5	>1024
1057	16	64	512	128	1024	1024	<0.5	<0.5	>1024
1075	8	8	512	64	32	64	<0.5	<0.5	1024
1065	8	128	512	256	1024	<0.5	<0.5	<0.5	>1024
1085	32	64	64	16	512	64	<0.5	<0.5	>1024
1053	16	256	512	128	>1024	1024	<0.5	<0.5	>1024
1054	32	256	256	128	>1024	1024	<0.5	<0.5	>1024
1013	16	16	64	32	512	16	<0.5	<0.5	>1024
1011	16	64	256	64	1024	1024	<0.5	<0.5	>1024
1034	16	64	256	128	256	128	<0.5	<0.5	>1024
1028	8	32	256	64	512	<0.5	<0.5	<0.5	>1024
1045	16	4	128	8	32	1024	<0.5	<0.5	1024
1118	32	32	128	32	256	1024	<0.5	<0.5	1024
1096	1	<0.5	>1024	<0.5	<0.5	<0.5	<0.5	<0.5	<0.5
1110	8	128	256	64	512	1024	<0.5	<0.5	>1024
1025	8	16	>1024	32	256	32	<0.5	<0.5	>1024
1016	16	16	128	64	256	16	<0.5	<0.5	>1024
4003	8	16	256	64	512	1024	<0.5	<0.5	>1024
90003	32	128	>1024	512	>1024	64	<0.5	<0.5	>1024
1020	128	<0.5	4	32	8	<0.5	<0.5	<0.5	>1024
1094	16	32	256	64	512	64	<0.5	<0.5	>1024
1019	8	128	>1024	512	>1024	64	<0.5	<0.5	>1024
1012	16	<0.5	<0.5	4	<0.5	32	<0.5	<0.5	8
2002	8	16	1024	32	256	32	<0.5	<0.5	>1024
4045	8	8	128	32	256	64	<0.5	<0.5	>1024
1081	8	32	256	32	512	1024	<0.5	<0.5	>1024
1018	4	32	128	64	1024	<0.5	<0.5	<0.5	>1024
3043	4	<0.5	<0.5	<0.5	<0.5	16	<0.5	<0.5	1024
1091	16	128	512	256	>1024	1024	<0.5	<0.5	>1024
3067	32	<0.5	16	64	64	<0.5	<0.5	<0.5	>1024
2003	16	64	256	64	512	4	<0.5	<0.5	>1024
1103	32	32	16	2	64	1024	<0.5	<0.5	>1024
1120	16	32	64	32	256	32	<0.5	<0.5	>1024
1119	8	64	256	64	1024	1024	<0.5	<0.5	>1024
1098	8	8	128	32	128	64	<0.5	<0.5	1024
1124	8	64	512	256	1024	32	<0.5	<0.5	>1024
1104	16	16	16	1	64	<0.5	<0.5	<0.5	>1024
1125	8	32	128	64	512	<0.5	<0.5	<0.5	>1024
1102	8	64	256	64	512	128	<0.5	<0.5	>1024
1117	16	128	256	128	1024	<0.5	<0.5	<0.5	>1024
1105	8	<0.5	<0.5	<0.5	<0.5	<0.5	<0.5	<0.5	>1024

### ESBL-producing *E. coli* genotyping

**Fig. 2:**
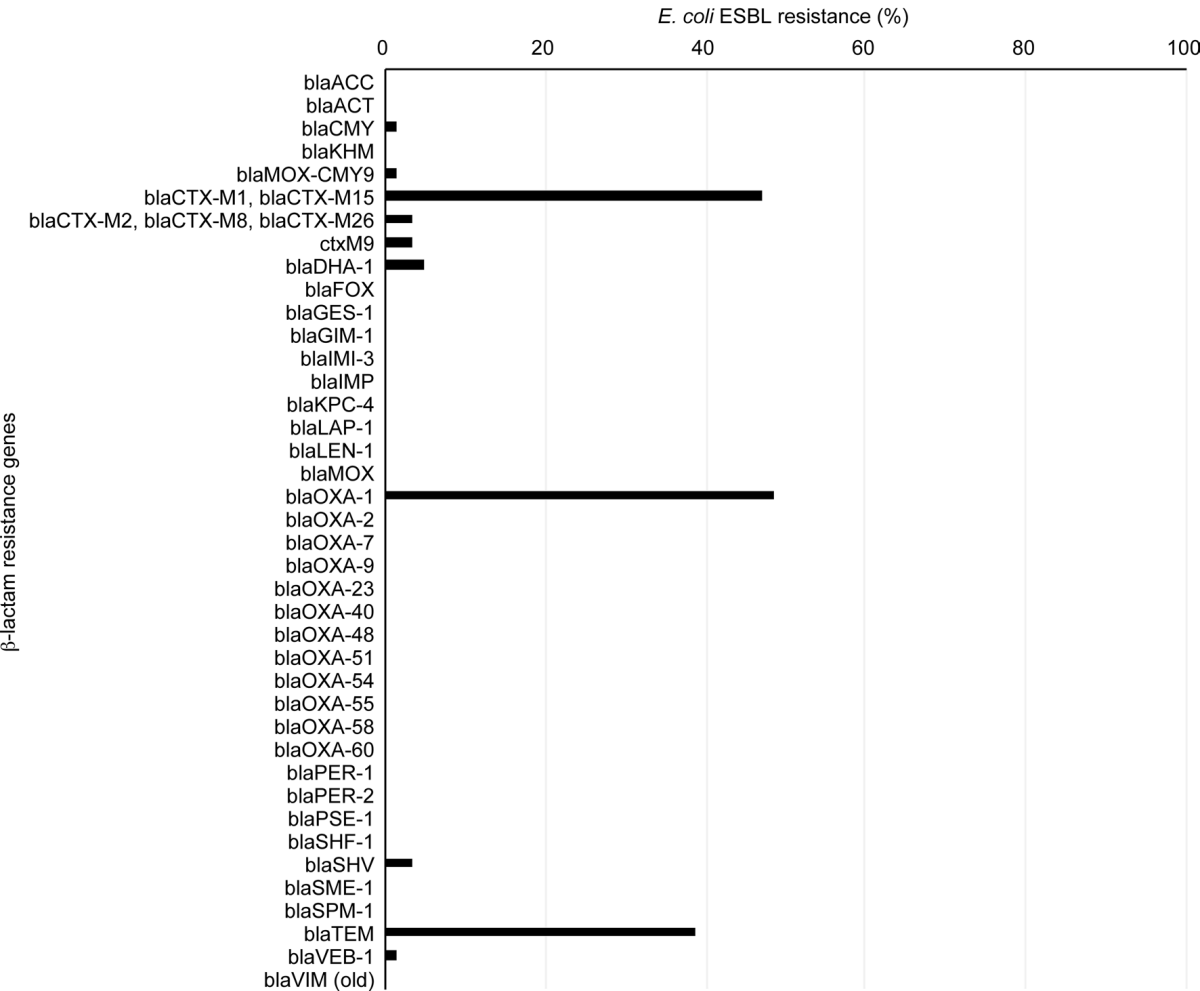
The distribution of the 58 ESBL-producing *E. coli* isolates investigated via oligonucleotide microarray-based assay and PCR genotyping against genes associated with β-lactam resistance

The genotyping of ESBL-producing *E. coli* isolates by the global analysis of an oligonucleotide microarray-based assay and PCR identified a high proportion of β-lactamase genes among the *E. coli* isolates. The *bla*CTX-M1, *bla*CTX-M15, blaOXA1 and *bla*TEM genotypes were more frequently identified and were more predominant among the *E. coli* isolates than the *bla*CTX-M, *bla*OXA and *bla*SHV variants. The prevalence was 46.7% for *bla*CTX-M1 and *bla*CTX-M15, 48.3% for *bla*OXA1, and 38.7% for *bla*TEM. The genotypic prevalence of *bla*SHV was 3.2% among all isolates, which was considerably lower than the prevalence for *bla*CTX-M1, *bla*CTX-M15, *bla*OXA1 and *bla*TEM. No carbapenem-resistant *E. coli* isolate was detected (Fig. 2).

### Distribution of ESBL genes in *E. coli*

The β-lactam genes were studied to evaluate the distribution of the genes among our isolates. We found that 13.7% (8/58) of the *E. coli* isolates harbored four major ESBL genes (bla_CTX-M1_, bla_CTX-M15_, bla_OXA1_, and bla_TEM_) and 17.2% (10/58) contained three ESBL genes (bla_CTX-M1_, bla_CTX-M15_, and bla_OXA1_). Additionally, two positive ESBL genotypes (3.4%; 2/58) contained bla_OXA1_ and bla_TEM_ and two isolates contained only bla_SHV_ (3.4%; 2/58). No carbapenemase genes were detected among the isolates according to the phenotypic and genotypic testing.

### The phylogenetic grouping of *E. coli* strains

**Fig. 3:**
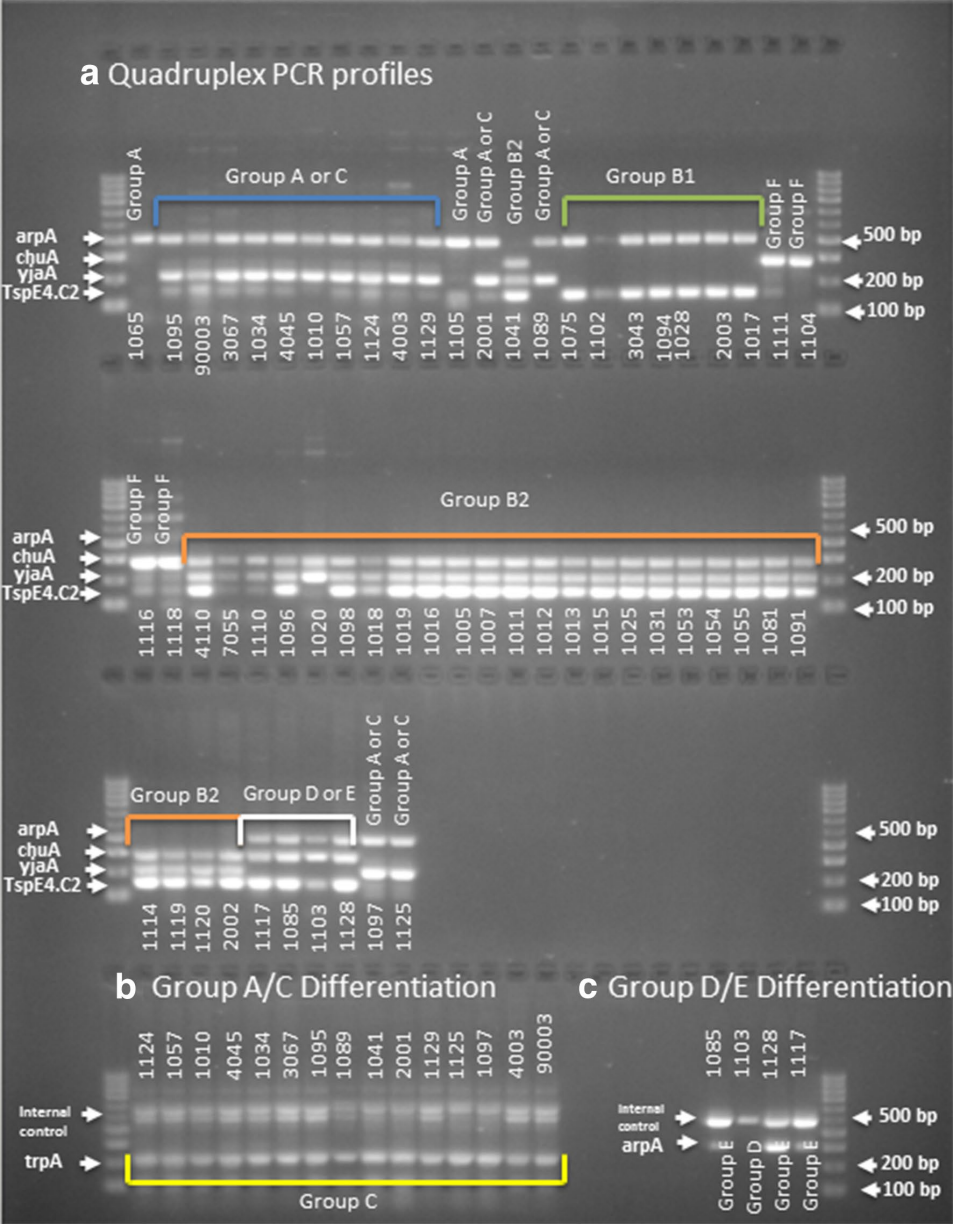
**a** Quadruples PCR profiles of 58 *E. coli* MDR isolates. Isolate IDs shown under *each lane*. Data assignment were performed according to the presence or absence of signals of the following gene order arpA, chuA, yjaA, TspE4.C2. *Row 1*, *lanes 1*, *12*—group A (+−−−); *lanes 2*–*11*, *13*, *15*—groups A or C (+−+−), *lanes 16*–*22*—group B1 (+−+), *lane 14*—group B2 (−+++), *lanes 23*–*24*—group F (−+−); *Row 2*, *lanes 1*–*2*—group F (−+−), *lanes 3*–*24*—group B2 (−+++) or (−++−) in *lane 7*; *Row 3*, *lanes 1*–*4*—group B2 (−+++), *lanes 5*–*8*—groups E or D (++−+), *lanes 9*–*10*—groups A or C (+−+−). **b** Groups A/C differentiation. In all *lanes*, both bands for internal control and Group C specific trpA fragment (219 bp) are present. **c** Groups D/E differentiation. *Lanes 1*, *3* and *4*—both bands for internal control and Group E specific arpA fragment (301 bp) are present. *Lane 2*—only internal control present

The genetic background grouping of the *E. coli* isolates was assigned based on the Clermont method which is very specific for *E. coli* phylo-typing groups [24]. It has shown that 46.55% (27) of the ESBL-producing *E. coli* isolate collection belonged to group B2, and 6.9% (4) belonged to group D or E, while 12% (7) belonged to group B1, 3.4% (2) belonged to group A, 24% (14) belonged to group A or C and 6.9% (4) belonged to group F. The majority of the pathogenic extra-intestinal *E. coli* strains belonged to groups B2 (Fig. 3).

### Prevalence of genes associated with aminoglycoside resistance in the *E. coli* isolates

**Fig. 4:**
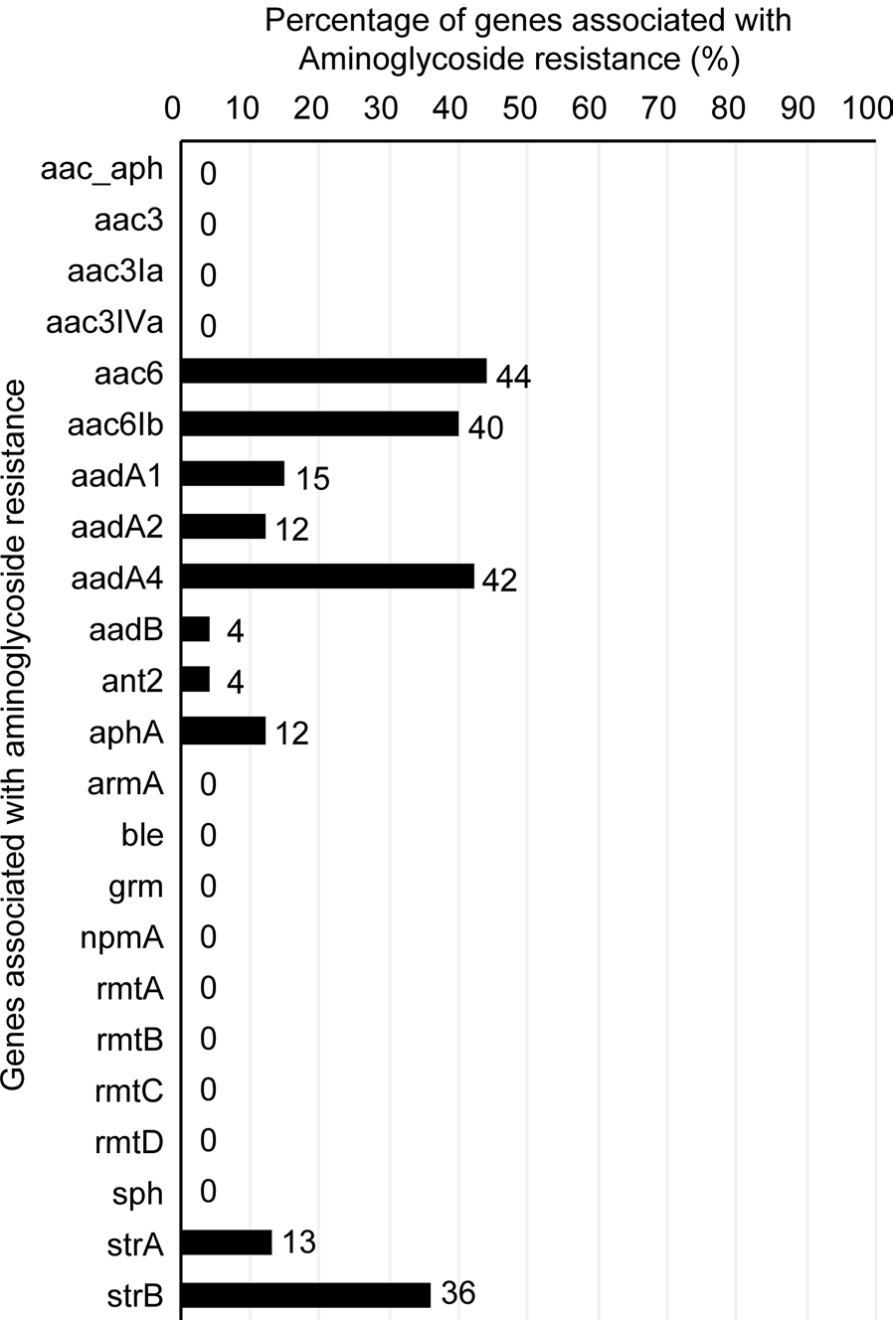
Prevalence of genes associated with aminoglycoside resistance

The prevalence of genes associated with aminoglycoside resistance among the 58 *E. coli* isolates was investigated. A total of 44.8% of the *E. coli* isolates carried the *aac6* gene, 43% harbored *aac6Ib*, 42% contained *aadA4*, and 36% contained *strB*; these prevalences represented high proportions and were more common than the other aminoglycoside genes (15% for *aadA1*, 12% for *aadA2*, 4% for *aadB*, 4% for *ant2*, 12% for *aphA* and 1% for *strA*) (Fig. 4).

## Bacterial genotyping by MLST

**Fig. 5:**
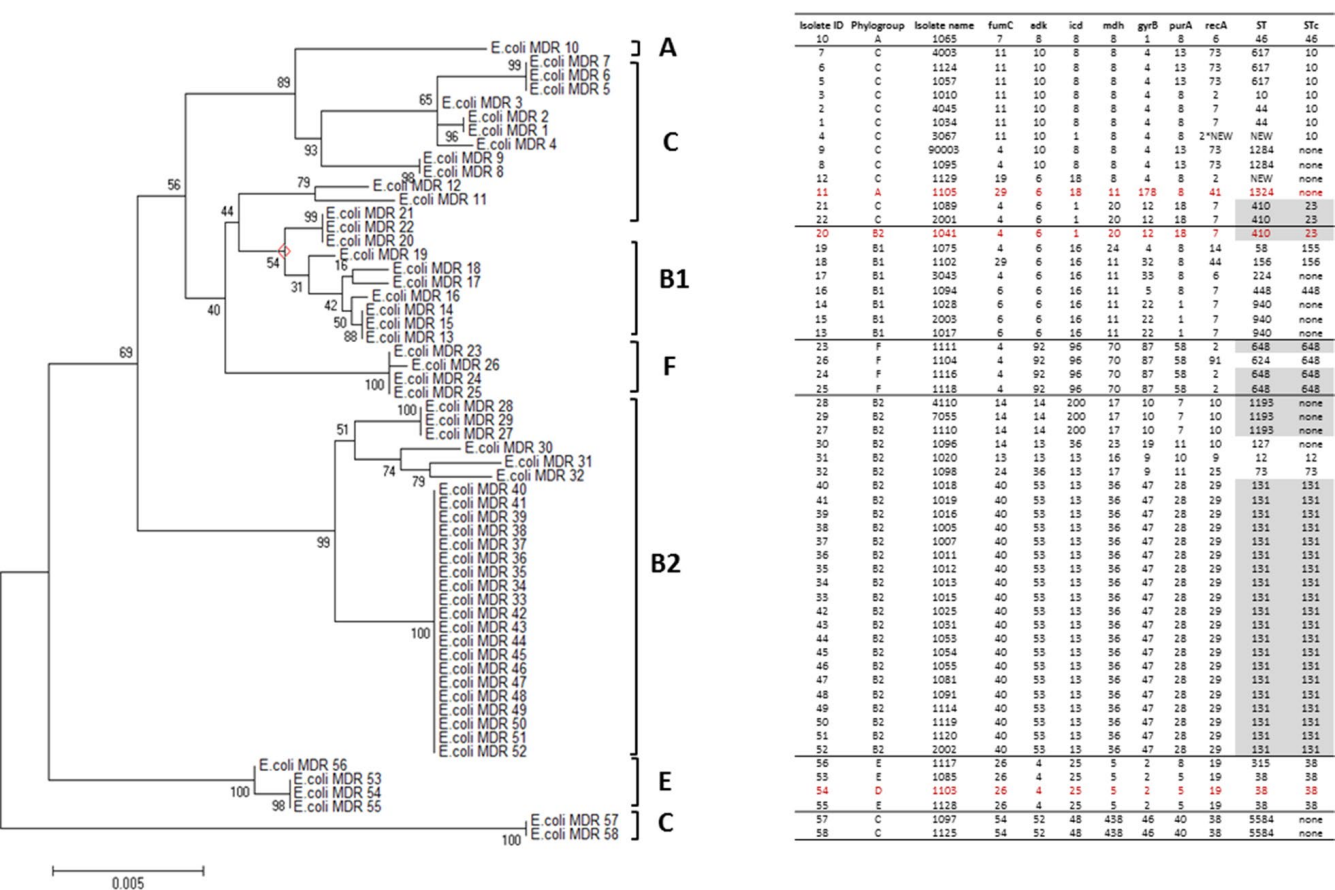
Phylogenetic tree of 58 MDR *E. coli* strains constructed using the maximum likelihood algorithm in the MEGA6 software [27] and based on the concatenated alleles of 7 housekeeping genes according to Achtman’s scheme (*columns 4*–*10*). The *numerals* on the branches represent bootstrap values. The phylogenetic analysis identified 24 ST (*column 11*) that corresponded to 11 ST complexes (STc, *column 12*). Phylogenetic groups in *column 2* were identified using phylo-typing method [24] by quadruplex PCR using four target genes (*arpA*±, *chuA*±, *yjaA*±, *TspE4.C2*±). Strains incorrectly assigned using the extended quadruplex method are indicated in *red*. The phylo-group memberships of two isolates (1097, 1125) were ambiguous. Notably, *E. coli* Clone ST131, ST410, and ST1193, which are disseminated globally, were among the identified STs in this study

In this study, 58 *E. coli* isolates were assigned sequence types (ST) and ST complexes (STc) using a standard multi-locus sequence typing method (Fig. 5) [25]. Of the 58 *E. coli* isolates, 7 isolates (12%) belonged to STc 10, 20 isolates (34.5%) to STc 131, and 4 isolates (7%) to STc 648 and STc 38. Other ST complexes (46, 448, 156, 155, 23, 12 and 73) were represented by 1–3 isolates (2–5%). The ST complex could not be determined for 14 isolates (24%). A new allele of the *rec*A gene was identified in isolate ID 4. This allele had one nucleotide substitution compared to allele 2 at position 40 from the beginning of the allele (C substituted by T). The allelic profile of one isolate (isolate ID 12) was not previously reported (Fig. 5).

## Phylogenetic analysis

We conducted a phylogenetic analysis to examine the clonal diversity and phylogenetic relationships among the *E. coli* isolates. The *E. coli* isolates clustered in several monophyletic clades that corresponded to known phylogenetic groups. The majority of the *E. coli* isolates fell into the phylogenetic groups B2: ST131 (20 isolates), ST1193 (3 isolates) [27], ST73 (1 isolate), ST12 (1 isolate) and ST127 (1 isolate). In this study, four *E. coli* isolates appeared to belong to group E: ST38 (3 isolates) [27] ST315 (1 isolate), of which all isolates were from STc 38. Of the rest, one isolate belonged to phylogroup A, thirteen to phylogroup C, eight to phylogroup B1, two to phylogroup C, and four to phylogroup F. Most of the known virulent extraintestinal strains primarily belong to group B2 and to a lesser extent to group D or E (4). Group A together with group B1, F, and C are considered predominant among commensal *E. coli* strains (5).

## Discussion

During congested visitation seasons, especially during the religious pilgrimage (Hajj), the risk of spreading multidrug-resistant microbes increases substantially [28]. Therefore, this study investigated the prevalence of ESBL-producing *E. coli* in local general hospitals in Makkah during Hajj and the correlation between the ESBL phenotype and antimicrobial drug resistance using genotyping approaches. This study also explored multilocus sequence typing (MLST) to characterize these isolates and to enhance our understanding of the epidemiology of ESBL-producing *E. coli* in our region. Our data had demonstrated that our *E. coli* isolates exhibited varying levels of resistance to ESBL drugs and carried genes associated with aminoglycoside resistance. No carbapenem resistance genes were identified in any of the collected and investigated *E. coli* isolates (n = 58). All uropathogenic *E. coli* isolates in this study are classified as multidrug-resistant (MDR) according to the criteria by Magiorakos [29].

The distribution of ESBL-producing *E. coli* was consistent with the data obtained from an oligonucleotide microarray-based assay and PCR genotyping against genes associated with β-lactam resistance. The dominant sequence type lineage of the isolates was ST131, which is the most uropathogenic *E. coli* lineage. The most commonly isolated bacterium from patients with urinary tract infections is *E. coli*. These isolates usually harbor different plasmids. Therefore, they have a tendency to acquire multidrug-resistant phenotypes and are difficult to treat.

Consistent with our study, studies have associated ST131/CTX-M15 human uropathogenic *E. coli* with ST410/CTX-M15, and ST648/CTX-M15. However, it was shown that ST410/CTX-M15, and ST648/CTX-M15 were also isolated from uropathogenic *E. coli* of cats and dogs (feline and canine) in Switzerland [30, 31]. These circulating STs may suggest that disease transmission between companion animals and humans may occur by direct contact. Furthermore, three isolates of *E. coli* ST1193 were evident in this study. *E. coli* ST1193 is associated with urinary tract infection and fluoroquinolone-resistance, namely ciprofloxacin-resistance (Figs. 1, 5) [32, 33]. *E. coli* ST1193 may also be transmitted through household contact as suggested by a study from Japan [34].

It was demonstrated that multidrug-resistance (MDR) *E. coli* clone ST131 was globally disseminated in six different geographical countries. The rapid spread and emergence among countries may be due to high virulence factor gene-content, the presence of ESBL CTX-M-15, and fluoroquinolones resistance. Notably, the *E. coli* clone ST131 fluoroquinolone resistance phenotype was the most prevalent geographically and was detected in this current study in Saudi Arabia (Figs. 1, 5) [35].

Studies have shown that pathogenicity island acquisition by uropathogenic *E. coli* may worsen urinary tract infections and aid in evasion of the host immune response, resulting in spreading in the bloodstream. Gene acquisition by intestinal pathogenic bacteria promotes their colonization in different intestinal regions; these genes may also alter the mechanism of bacterial-host interactions, causing distinct gastrointestinal pathology [27]. It was shown that a urinary sepsis niche for *E. coli* ST131 compared to bacteremia in non-ST131 *E. coli* clones [32, 36]. However, this niche could not be linked to the clinical manifestation of renal tract infections in humans. No clinical focus to other sites of infection, such as intra-abdominal abscess, ascitic fluid, bones/joints, respiratory tract or bacteremia, has been established for the ST131 clone [37, 38]. There is evidence for a direct human-to-human route of transmission for ST131. For instance, an elderly father with pyelonephritis transmitted ST131 *E. coli* to his daughter, which initiated a similar illness in her. Similarly, an identical clone was identified from a young child with osteoarticular infection and a fecal sample from his mother [39, 40]. ST131 may contain genes for multiple antimicrobial resistance mechanisms, which may make therapy difficult. Carbapenems alone or in combination with amikacin were successfully used to treat infections caused by clones harboring CTX-M genes [32, 39]. A study demonstrated ESBL-producing *E. coli* acquisition among Makkah visitors after the Hajj (pilgrimage) season, especially CTX-M genes [28].

Molecular epidemiological studies have focused on the characterization and spread of the ST131 clone; however, global studies on the distribution and spread between humans and animals are limited, particularly in the developing world. There is a high frequency of infection by the drug-resistant ST131 clone in developing countries and a lack of timely countermeasures resulting in high morbidity and mortality rates [41–43].

## Conclusion

In this study, 58 *E. coli* isolates were collected from patients with urinary tract infections from two local hospitals in the city of Makkah during the 2014–2015 Hajj (pilgrimage) season and screened for the production of ESBL/AmpC/carbapenemase/aminoglycoside resistance genes using phenotypic and genotypic analyses. Our data had demonstrated that our isolates primarily carried various ESBL and aminoglycoside resistance genes. The dominant sequence type lineage of the isolates was ST131, which was the most uropathogenic *E. coli* lineage. A genetic analysis of multiple isolates from the Middle Eastern regions may contribute to the development of rapid molecular detection methods and may lead to new therapies. Furthermore, controlling the endemicity of emerging MDR and decreasing the occurrence and prevalence of drug-resistant uropathogenic *E. coli* during heavy visitation seasons in the city of Makkah may involve the implementation of a stringent antidrug treatment policy.

## Abbreviations

MDR: multi drug resistance
ESBL: extended-spectrum-β-lactamase
AmpC: AmpC beta-lactamases
MIC: minimum inhibitory concentration
MLST: multilocus sequence typing
ST: sequence typing
STc: sequence typing complex
